# Prognostic value of early in-hospital glycemic excursion in elderly patients with acute myocardial infarction

**DOI:** 10.1186/1475-2840-12-33

**Published:** 2013-02-11

**Authors:** Gong Su, Shu-hua Mi, Zhao Li, Hong Tao, Hong-xia Yang, Hong Zheng

**Affiliations:** 1 Department of Cardiology, Beijing Anzhen Hospital of Capital Medical University, No. 2 Anzhen Road, Chaoyang district, Beijing, China; 2 Beijing Emergency Center of Heart, Lung & Blood Vessel Diseases, Beijing, China; 3 Department of Endocrinology, Beijing An Zhen Hospital of Capital Medical University, Beijing, China

## Abstract

**Background:**

Acute phase hyperglycemia has been associated with increased mortality in patients with acute myocardial infarction (AMI). However, the predictive value of glycemic excursion for adverse outcome in elderly AMI patients is not clear. The aim of this study is to investigate the prognostic value of early in-hospital glycemic excursion and hemoglobin A_1c_ (HbA_1c_) for one-year major adverse cardiac event (MACE) in elderly patients with AMI.

**Methods:**

We studied 186 elderly AMI patients, whose clinical data were collected and the Global Registry of Acute Coronary Events (GRACE) risk score were calculated on admission. The fluctuations of blood glucose in patients were measured by a continuous glucose monitoring system (CGMS) for 72 hours. Participants were grouped into tertiles of mean amplitude of glycemic excursions (MAGE) and grouped into HbA_1c_ levels (as ≥6.5% or <6.5%). The MACE of patients, including new-onset myocardial infarction, acute heart failure and cardiac death, was documented during one year follow-up. The relationship of MAGE and HbA_1c_ to the incidence of MACE in elderly AMI patients was analyzed.

**Results:**

In all participants, a higher MAGE level was associated with the higher GRACE score (r = 0.335, p < 0.001). The rate of MACE by MAGE tertiles (>3.94 mmol/L, 2.55-3.94 mmol/L or <2.55 mmol/L) was 30.2% vs. 14.8% vs. 8.1%, respectively (p = 0.004); by HbA_1c_ category (≥6.5% vs. <6.5%) was 22.7% vs. 14.4%, respectively (p = 0.148). Elderly AMI patients with a higher MAGE level had a significantly higher cardiac mortality. In multivariable analysis, high MAGE level was significantly associated with incidence of MACE (HR 3.107, 95% CI 1.190-8.117, p = 0.021) even after adjusting for GRACE risk score, but HbA_1c_ was not.

**Conclusions:**

The early in-hospital intraday glycemic excursion may be an important predictor of mortality and MACE even stronger than HbA_1c_ in elderly patients after AMI.

## Background

Increasing age is considered one of the most significant risk factors for acute myocardial infarction (AMI) [1]. Ageing is also a risk factor that contributes to variance in diabetes risk [2]. Dysglycemia is associated with poor outcomes in AMI patients both with and without diabetes. Chronic glucose dysregulation, as assessed by haemoglobin A_1c_ (HbA_1c_) levels, is a prognostic factor for mortality in patients with AMI [3,4]. It is evident that admission hyperglycemia is of independent prognostic value with regard to future adverse cardiovascular events in patients with AMI [5,6]. Recent studies have showed that glycemic excursion might play an important role in the pathogenesis of atherosclerosis and may be an independent risk factor for cardiovascular complications in diabetics [7,8]. However, it still remains unclear whether acute glycemic excursion has the important prognostic significance in elderly AMI patients. The purpose of the current study is therefore to investigate the independent prognostic value of glycemic excursion determined by a continuous glucose monitoring system (CGMS) and HbA_1c_ levels in elderly patients with AMI.

## Methods

### Study population

Consecutive patients admitted to the cardiology department of Beijing An Zhen Hospital of Capital Medical University for AMI between July 2010 and October 2011 were selected. The inclusion criteria were: (i) age ≥ 60 years old; (ii) confirmed admission diagnosis of AMI including ST segment elevated myocardial infarction (STEMI) and non-ST segment elevated myocardial infarction (NSTEMI); (iii) admission glucose < 16.7 mmol/L, and without diabetic ketosis or nonketotic hyperosmolar coma. AMI was defined as acute if the time elapsed between the first symptom and admission was 72 h or less. To enable long-term follow-up and repeated visits to our outpatient clinic, only patients under the age of 80 and living within the hospital’s catchment area were eligible. The exclusion criteria were severe non-cardiac disease with expected survival of less than one year and unwillingness to participate. A patient could only be included once. Data, including information on previous clinical history, cardiovascular risk factors and medication, were collected in hospital. Type 2 diabetes mellitus (T2DM) was diagnosed according to the American Diabetes Association criteria or the use of insulin or glucose-lowering medication. The estimated glomerular filtration rate (eGFR) value was calculated by MDRD equation [9]. The Global Registry of Acute Coronary Events (GRACE) risk score were calculated as admission [10], which is recommended by the National Institute for Health and Clinical Excellence (NICE) to assess risk in patients with ACS. Patients were categorized according to tertiles of the mean amplitude of glycemic excursions (MAGE) level (<2.55 mmol/L, 2.55-3.94 mmol/L and >3.94 mmol/L), and according to HbA_1c_ (<6.5% and ≥6.5%) [11]. The study protocol was approved beforehand by the Medical Ethics Committee of Beijing An Zhen Hospital of Capital Medical University and the procedures followed were in accordance with the institutional guidelines. The study complied with the declaration of Helsinki and informed consent was obtained from all patients.

### Continuous glucose monitoring

All patients were equipped with CGMS (Medtronic MiniMed, USA), and were monitored for 72 consecutive hours after admission. A CGMS sensor was inserted into the subcutaneous abdominal fat tissue, calibrated according to the standard Medtronic MiniMed operating guidelines. During CGMS monitoring, patients checked their blood glucose level with a self-monitoring of blood glucose (SMBG) device (Medisafe Mini, Terumo, Japan) at least 4 times per day. Then, they entered the SMBG data and time of each meal into the CGMS. After monitoring for 72 hours, the recorded data were downloaded into a personal computer for analysis of the glucose profile and glycemic excursion parameters with MiniMed Solutions software. After downloading the recorded data, the intermediate 48 hours of recording was analyzed to avoid bias due to insertion and removal of the CGMS or insufficient stability of the monitoring system, and MAGE was caculated from the first 24 hours of them. Since measurable range of glucose by CGMS was mechanically limited from 2.2 to 22.2 mmol/L, the case showing the data out of this range was excluded from the study. The MAGE was calculated by measuring the arithmetic mean of the differences between consecutive peaks and nadirs, provided that the differences are greater than one standard deviation (SD) of the mean glucose value [12]. Patients would maintain anti-hyperglycemic therapy as usual and be avoided glucose infusion during CGMS monitoring period. Otherwise, the patient would be excluded from the study.

### Biochemical investigations

Blood samples were collected after overnight fasting and stored at −70°C prior to analysis. Serum creatinine, total cholesterol (TC) and triglyceride (TG) levels were measured by automatic biochemical analyzer (Hitachi 747, Tokyo, Japan). Serum concentration of hemoglobin A_1c_ (HbA_1c_) was determined by high-performance liquid chromatographic method using automatic HbA_1c_ analyzer (Tosoh HLC-723 G7, Japan).

### Follow-up

Patients were followed up prospectively for about 1 year. During follow-up period, incidences of major adverse cardiac event (MACE) were registered, including new-onset myocardial infarction, acute heart failure and cardiac death. All MACE data were adjudicated by an experienced cardiovascular physician blinded to clinical details and outcomes.

### Statistical analysis

All statistical analyses were performed by using SPSS for Windows 13.0 (SPSS Inc, Chicago, IL, USA). Data are presented as frequencies and percentages for categorical variables and mean ± SD for continuous variables, unless otherwise indicated. Differences between two groups were assessed by using the Chi-square and unpaired *t*-tests. Correlation between continuous variables was determined by Spearman correlation coefficients. MAGE was included as a continuous and as a categorized (<2.55 mmol/L, 2.55-3.94 mmol/L and >3.94 mmol/L) variable. HbA_1c_ level was also included as a continuous and categorized (<6.5% and ≥6.5%) variable. Kaplan-Meier survival curve analysis was used to represent the proportional risk of MACE for the MAGE and HbA_1c_ values, and the log-rank test was performed to assess differences between high MAGE level and low MAGE level, and high HbA_1c_ level and low HbA_1c_ level. Cox proportional-hazards regression models were used to estimate hazard ratios of clinical variables with regard to MACE. A value of *p* < 0.05 was considered statistically significant.

## Results

### Baseline characteristics

During the study period, 200 elderly AMI patients were enrolled. 186 patients with complete data were included in the final analysis (8 patients were removed from study for severe dysglycemia during CGMS monitoring period; 6 patients were excluded from study for incomplete follow-up data). Mean age was 67.0 ± 5.7 years, 60.4% were male and 54.3% had diabetes. Participants were treated conservatively (9.1%), with PCI (80.1%) or with CABG (10.8%). HbA_1c_ was < 6.5% in 111 (59.7%), ≥ 6.5% in 75 (40.3%). The GRACE risk score ranged from 78 to 235 with a mean of 148 ± 36. Baseline characteristics of patient groups based on MAGE and HbA_1c_ are shown in Table 1 and 2, respectively. The correlation of GRACE score with MAGE or HbA_1c_ was significant (Spearman r = 0.335, p < 0.001; r = 0.188, p = 0.010).

**Table 1:** Baseline characteristics in AMI patients according to MAGE

**MAGE (mmol/L)**	**< 2.55**	**2.55-3.94**	**> 3.94**
n	62	61	63
Age (years)	65.5 ± 5.2	67.1 ± 5.0	68.4 ± 6.4 *
Males	43 (69.4)	34 (55.7)	39 (61.9)
**Risk factors**			
Diabetes	22 (35.5)	32 (52.5)	47 (74.6) *#
Previous CAD	14 (22.6)	15 (24.6)	29 (46.0) *#
Smoking	22 (35.5)	18 (29.5)	34 (54.0) *#
BMI (kg/m^2^)	25.9 ± 2.4	26.5 ± 2.7	26.8 ± 3.3
Systolic BP (mmHg)	125 ± 18	127 ± 18	130 ± 23
Diastolic BP (mmHg)	75 ± 10	77 ± 9	78 ± 11
Heart rate (bpm)	68 ± 12	73 ± 12 *	72 ± 11 *
LVEF (%)	58.2 ± 10.5	57.2 ± 13.4	51.4 ± 11.1 *#
eGFR (ml/min/1.73 m^2^)	76.2 ± 30.3	64.2 ± 26.5 *	62.5 ± 20.8 *
TC (mmol/L)	4.46 ± 0.99	4.55 ± 1.02	4.62 ± 1.15
TG (mmol/L)	2.13 ± 1.04	1.96 ± 0.99	2.25 ± 1.78
FBG (mmol/L)	6.53 ± 1.96	7.41 ± 2.29 *	8.31 ± 2.98 *
HbA_1c_ (%)	5.8 ± 0.9	6.4 ± 1.2 *	7.1 ± 1.2 *
**Medications**			
Aspirin	58 (93.5)	55 (90.1)	60 (95.2)
Beta-blocker	24 (38.7)	24 (39.3)	30 (47.6)
Oral anti-hyperglycemic	19 (30.6)	22 (36.1)	26 (41.3)
Insulin	8 (12.9)	22 (36.1) *	33 (52.4) *
ACEI	25 (40.3)	30 (49.2)	32 (50.8)
Statin	48 (77.4)	47 (77.0)	51 (80.9)
Diuretic	9 (14.5)	11 (18.0)	17 (27.0)
**Type of AMI**			
STEMI	37 (59.7)	49 (80.3) *	48 (76.2) *
Non-STEMI	25 (40.3)	12 (19.7) *	15 (23.8) *
GRACE Score	128 ± 29	144 ± 29 *	155 ± 39 *

Abbreviations: MAGE, the mean amplitude of glycemic excursions; CAD, coronary artery disease; BMI, body mass index; BP, blood pressure; LVEF, left ventricular ejection fraction; eGFR, estimated glomerular filtration rate; TC, total cholesterol; TG, triglyceride; FBG, fasting blood glucose; HbA_1c_, hemoglobin A_1c_; ACEI, angiotensin converting enzyme inhibitor; AMI, acute myocardial infarction; STEMI, ST segment elevated myocardial infarction; GRACE, the global registry of acute coronary events. * p < 0.05 vs. MAGE < 2.55 mmol/L; # p < 0.05 vs. MAGE 2.55-3.94 mmol/L. Data are mean ± SD and number (%).

**Table 2:** **Baseline characteristics in AMI patients according to HbA**_**1c **_**level**

**HbA**_**1c **_**(%)**	**< 6.5**	**≥ 6.5**	**P**
n	111	75	
Age (years)	66.0 ± 5.1	68.6 ± 6.2	0.002
Males	67 (60.4)	49 (65.3)	0.492
**Risk factors**			
Diabetes	49 (44.1)	52 (69.3)	0.001
Previous CAD	30 (27.0)	28 (37.3)	0.137
Smoking	41 (36.9)	33 (44.0)	0.334
BMI (kg/m^2^)	26.3 ± 2.8	26.5 ± 3.0	0.556
SBP (mmHg)	126 ± 18	128 ± 21	0.435
DBP (mmHg)	76 ± 10	77 ± 13	0.720
HR (bpm)	70 ± 11	72 ± 13	0.234
LVEF (%)	57.8 ± 11.6	53.2 ± 11.9	0.010
eGFR (ml/min/1.73 m^2^)	70.6 ± 31.8	63.2 ± 25.7	0.061
TC (mmol/L)	4.45 ± 0.97	4.67 ± 1.15	0.167
TG (mmol/L)	1.96 ± 1.00	2.34 ± 1.68	0.056
FBG (mmol/L)	6.58 ± 1.73	9.12 ± 2.63	< 0.001
MAGE (mmol/L)	2.64 ± 1.23	4.15 ± 1.28	< 0.001
**Medications**			
Aspirin	101 (91.0)	72 (96.0)	0.189
Beta-blocker	46 (41.4)	32 (42.7)	0.868
Oral anti-hyperglycemic	24 (21.6)	43 (57.3)	< 0.001
Insulin	17 (15.3)	46 (61.3)	< 0.001
ACEI	51 (45.9)	36 (48.0)	0.783
Statin	85 (76.6)	61 (81.3)	0.439
Diuretic	13 (11.7)	24 (32.0)	0.001
**Type of AMI**			
STEMI	74 (66.7)	60 (80.0)	0.047
Non-STEMI	37 (33.3)	15 (20.0)	0.047
GRACE Score	137 ± 32	151 ± 36	0.005

Abbreviations: HbA_1c_, hemoglobin A_1c_; CAD, coronary artery disease; BMI, body mass index; BP, blood pressure; LVEF, left ventricular ejection fraction; eGFR, estimated glomerular filtration rate; TC, total cholesterol; TG, triglyceride; FBG, fasting blood glucose; MAGE, the mean amplitude of glycemic excursions; ACEI, angiotensin converting enzyme inhibitor; AMI, acute myocardial infarction; STEMI, ST segment elevated myocardial infarction; GRACE, the global registry of acute coronary events. Data are mean ± SD and number (%).

### Incidences of MACE

**Figure 1:**
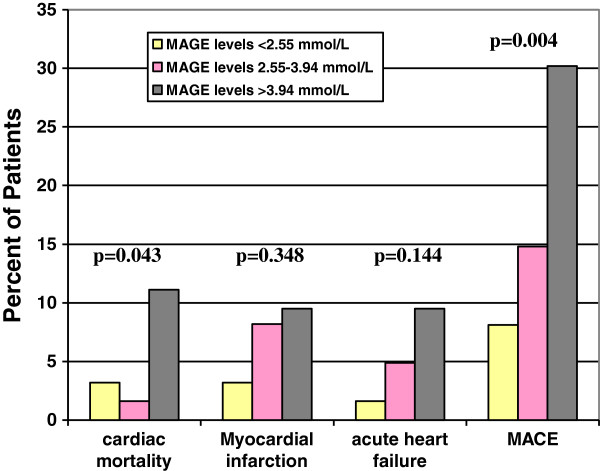
**Incidence of MACE after 1-year follow-up in relation to MAGE levels.** Elderly AMI patients with a higher MAGE level had significantly higher cardiac mortality and incidence of all MACE

**Figure 2:**
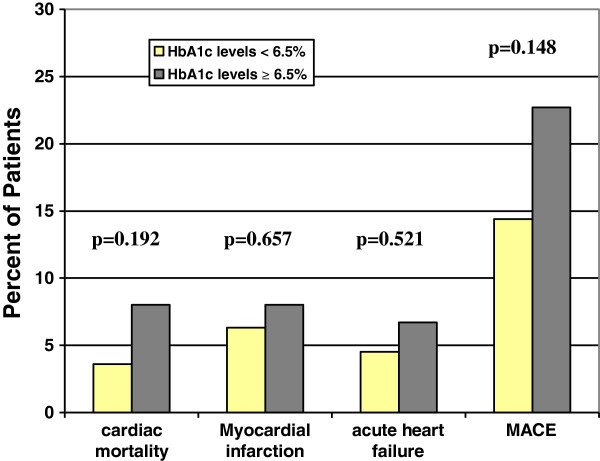
**Incidence of MACE after 1-year follow-up in relation to HbA**_**1c **_**levels.** There are no significant differences of adverse cardiovascular events rates between different HbA_1c_ level groups (all p > 0.05)

**Figure 3:**
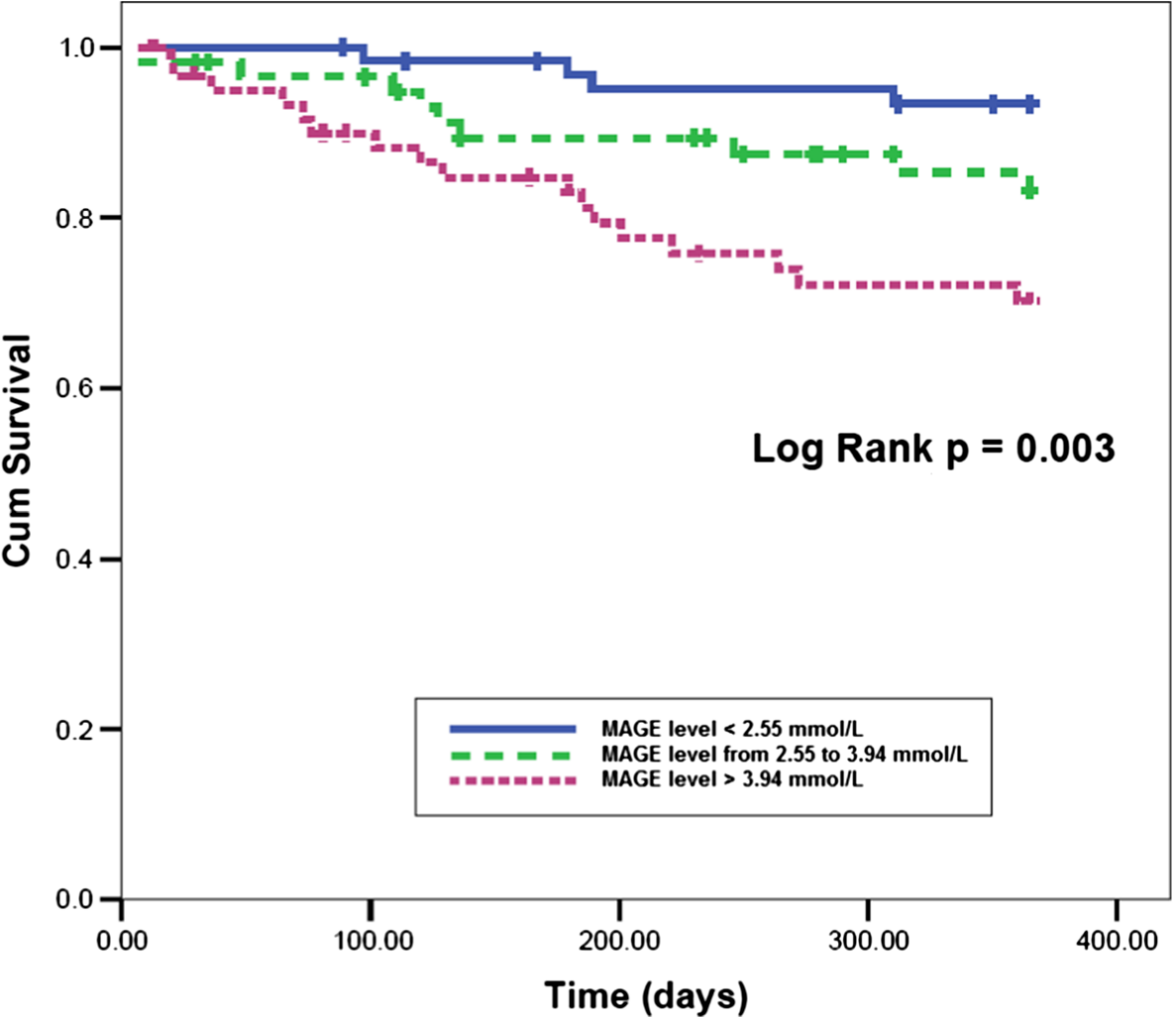
**Kaplan-Meier event-free survival curves for freedom from MACE in three patient groups by MAGE levels.** The event-free survival rate was significantly lower in the high MAGE level group (log-rank test, p = 0.003)

**Figure 4:**
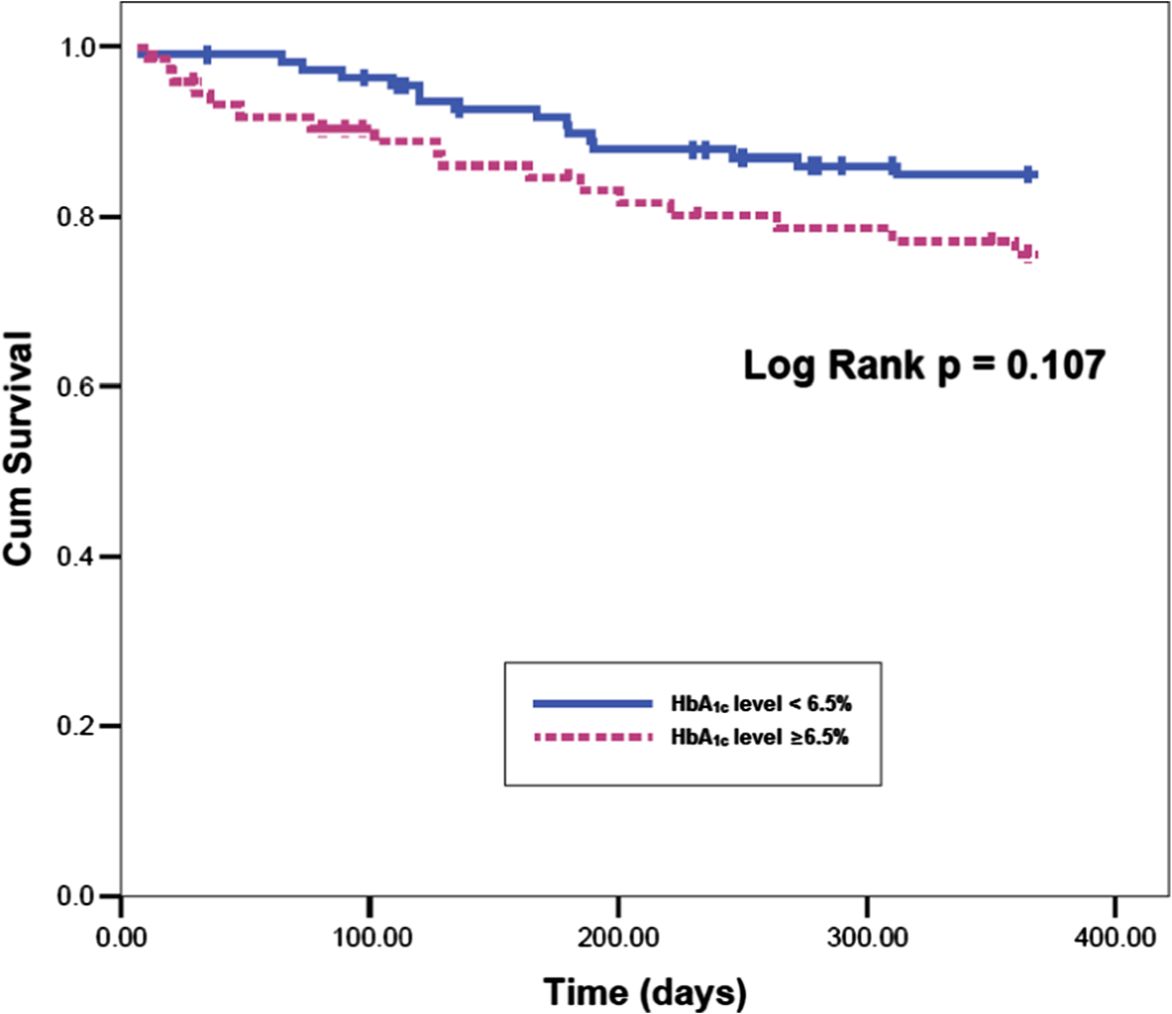
**Kaplan-Meier event-free survival curves for freedom from MACE in two patient groups by HbA**_**1c **_**levels.** There is not significant lower event-free survival rate in high HbA_1c_ level patients (log-rank test, p = 0.107)

At the end of 1-year follow-up, 10 patients had died (5.4%) for cardiac causes, 13 patients had new-onset myocardial infarction (7.0%), and 10 patients had acute heart failure (5.4%). As expected, elderly AMI patients with MAGE level >3.94 mmol/L had significantly higher incidence of MACE compared with elderly AMI patients with MAGE level from 2.55 mmol/L to 3.94 mmol/L, or < 2.55 mmol/L (30.2% vs. 14.8% vs. 8.1%, p = 0.004). No significant rates of adverse cardiovascular events were observed between patients with HbA_1c_ level ≥6.5% and patients with HbA_1c_ level < 6.5% (22.7% vs. 14.4%, p = 0.148). Elderly AMI patients with a higher MAGE level had a significantly higher cardiac mortality compared with elderly AMI patients with lower MAGE levels (11.1% vs. 1.6% vs. 3.2%, p = 0.043) (Figure 1). No significant differences in rates of adverse cardiovascular events were observed between elderly AMI patients with high HbA_1c_ level and patients with low HbA_1c_ level (Figure 2). Kaplan-Meier survival curves for patient groups by MAGE are shown in Figure 3; those for HbA_1c_ in Figure 4.

### Multivariable analysis

To investigate the associations between MAGE, HbA_1c_ level and incidences of MACE with respect to baseline characteristics, we used multivariable analysis. Include variables were: age, gender, and all variables that were significantly different between MAGE or HbA_1c_ categories [current smoking, diabetes, previous coronary artery disease (CAD), heart rate, eGFR, anti-hyperglycemic agents, fasting blood glucose, the STEMI presentation, left ventricular ejection fraction (LVEF, categorized into <50% = 1 and ≥50% = 0) and diuretics]. The independent predictors of MACE were: age (HR 1.624, 95% CI 1.036-2.548, p = 0.035), previous CAD (HR 2.907, 95% CI 1.227-6.896, p =0.015), LVEF (HR 2.611, 95% CI 1.107-6.135, p = 0.028) and MAGE (HR 3.131, 95% CI 1.422-6.710, p = 0.014). HbA_1c_ level was not significantly associated with MACE (HR 1.522, 95% CI 0.841-2.757, p = 0.165). After adjustment for the GRACE score, MAGE was found to be also associated with incidences of MACE (HR 3.107, 95% CI 1.190-8.117, p = 0.021), but HbA_1c_ was not (HR 1.438, 95% CI 0.619-5.464, p = 0.273).

## Discussion

Hyperglycemia on admission is common in patients with AMI, and it is a powerful predictor of survival and increased risk of MACE in patients both with and without T2DM [3,5,6]. HbA_1c_ is a convenient marker of long-term glycometabolic status. Elevated HbA_1c_ is associated with increased cardiovascular risk in patients. However, patients with similar mean glucose or HbA_1c_ levels can have markedly different glycemic excursions [13]. Acute glucose fluctuations seem to have more deleterious effects than sustained hyperglycemia in the development of cardiovascular complications as glucose both upward and downward changes activate the oxidative stress [14,15]. We investigated the association between glycemic excursion, HbA_1c_ and one-year MACE in elderly patients with AMI. Our study demonstrated that elevated MAGE was a strong and independent predictor of increased risk of MACE in elderly patients with AMI, but HbA_1c_ was not.

There were major differences in baseline characteristics according to MAGE or HbA_1c_ level. Patients with higher MAGE or HbA_1c_ level had more cardiovascular risk factors, such as older age, diabetes, heart failure or renal insufficiency. There was also a clear correlation between GRACE risk scores and MAGE or HbA_1c_. The results indicate that elderly AMI patients with worse glycometabolic disorders may be associated with poorer outcomes.

More and more evidences show that glycemic variability may be an important parameter used to resolve potential clinical problems in diabetes. It is reported that postchallenge glucose excursion is independently related to carotid intima-media thickness and may contribute to the development of atherosclerosis in individuals with T2DM independent of other risk factors [7,16]. In our previous study, we found that glycemic variability is an important contributing factor in the severity of coronary artery disease, which is independent of the average level of blood glucose [8]. The Verona Diabetes study reported that fasting glycemic variability is an independent predictor of mortality in T2DM patients [17]. Some studies concluded that glycemic excursion was a significant predictor of mortality in critically ill patients independently from mean glucose level and severity of illness [18,19]. In the present study, patients with a higher MAGE level have higher GRACE risk scores. After 1-year follow-up, a significantly higher incidence of MACE and cardiac mortality were found in those patients. The results indicate that high glucose fluctuations may be associated with the risk of future adverse cardiovascular events in patients with AMI. Multivariable analysis disclosed that in the elderly AMI population, MAGE was an independent predictor of MACE, even after adjusting for GRACE risk score, but HbA_1c_ was not.

Acute hyperglycaemia is a common acute adrenergic signal of stress and is present in myocardial infarction, whereas increased catecholamine levels result in decreased insulin secretion and increased insulin resistance [20]. Although stress-induced hyperglycaemia can partly explain the relation between admission glycemic variability and outcomes, glycemic excursion itself can also be harmful. Ceriello et al. reported that intermittent hyperglycaemia induced a higher degree of apoptosis in endothelial cells than chronic hyperglycaemia [14]. Quagliaro et al. showed that the apoptosis of endothelial cells exposed to intermittent high glucose may be related to a reactive oxygen species (ROS) overproduction, through protein kinase C (PKC)-dependent activation of nicotinamide adenine dinucleotide phosphate (NADPH)-oxidase [21]. Glycemic excursion may also be an important mediator in inflammatory responses. *In vitro* studies indicate that glucose fluctuations can activate nuclear factor-κB and PKC pathway, leading to a greater expression of the adhesion molecules and excess formation of advanced glycation end-products than stable high glucose [22]. Moreover, severe glycemic disorders may adversely affect sympathetic dysfunction which is associated with mortality and morbidity of cardiovascular disease [23].

Although both HbA_1c_ and glycemic excursion may be associated with adverse prognosis, our study show that increased MAGE is more important. In our analysis, the less clear association between HbA_1c_ and MACE could be due to a limited number of patients with a relatively short follow-up in present study. Increased HbA_1c_ represents long-term glucose regulation, whereas elevated glycemic excursion is not only a symptom of glucose dysregulation, but also of stress and general poor health. Carmen Wong et al. found cortisol level is correlated with acute hyperglycaemia in patients with AMI [24]. There is a clear association to be found between HbA_1c_ and long-term outcome in AMI patients after 3.3 years follow-up [25]. These differences may relate to length of follow-up. HbA_1c_ may have limited predictive power for short-term prognosis in patients with AMI, but its association with long-term prognosis may be stronger.

There is still an extensive debate about glycemic excursion as a risk factor for cardiovascular complications independent of HbA_1c_[26,27]. Siegelaar et al. performed reanalysis of the data of the HEART2D, which shows that targeting post-prandial glucose decreased intraday glycemic excursion would not be beneficial in reducing adverse cardiovascular events in AMI patients [28]. However, the HEART2D was not designed to determine the impact of glycemic excursion on the risk of MACE, and the MAGE and SD levels were not found significantly different between two contrasting groups in the study. In addition, the method of calculating glycemic excursion from self-measured blood glucose profiles may be not very accurate. Overall, more well-designed studies are needed to investigate whether glycemic excursion will play an important role in the prognosis of AMI.

### Study limitations

The sample size was relatively small, so that comparisons of some subgroups might lack power to detect significant differences for selected variables. For lack of microvascular complications data, we didn’t include those risk factors in analysis. Although we had maintained the patients’ anti-hyperglycemic therapy as usual and avoided glucose infusion during CGMS monitoring period, some factors, such as different diets, physical and emotional stress etc., which may affect glucose fluctuations couldn’t be all prevented. In addition, tests to detect diabetes were not routinely done, so some cases of diabetes may have been missed. However, if the observed relation between glycemic excursion and MACE was due to undiagnosed diabetes, one would have expected a more distinct association between HbA_1c_ and outcomes.

## Conclusions

In elderly patients, early in-hospital MAGE may be an important predictor of mortality and MACE after AMI even stronger than HbA_1c_. The results of this study further support the view that glycemic excursion should be one of the targets of treatment for the glycemic disorders encountered in AMI patients. Further studies are needed to determine if pharmacologic therapy aimed at controlling glucose excursion in AMI would be beneficial in prognosis of this high-risk patient population.

## Competing interests

The authors declare that they have no competing interests.

## Authors’ contributions

GS participated in the design of the study, participated in the exercise protocols, performed the statistical analysis and drafted the manuscript. SHM and HT participated in the design of the study and drafted the manuscript. ZL, HXY and HZ participated in the exercise protocols. All authors approved the final manuscript.
